# Profiling Real-Time Aroma from Green Tea Infusion during Brewing

**DOI:** 10.3390/foods11050684

**Published:** 2022-02-25

**Authors:** Litao Sun, Xue Dong, Yonglin Ren, Manjree Agarwal, Alexander Ren, Zhaotang Ding

**Affiliations:** 1 Tea Research Institute, Qingdao Agricultural University, Qingdao 266109, China; slttea@163.com; 2 College of Science, Health, Engineering and Education, Murdoch University, 90 South Street, Perth, WA 6150, Australia; xue_dong1990@126.com (X.D.); y.ren@murdoch.edu.au (Y.R.); m.agarwal@murdoch.edu.au (M.A.); alex_ren97@hotmail.com (A.R.); 3 Institute of Agricultural Resources and Environment, Jiangsu Academy of Agricultural Sciences, 50 Zhongling Street, Nanjing 210014, China

**Keywords:** green tea, tea aroma, real-time aroma, tea brewing, HS-SPME, DI-SPME, VOCs

## Abstract

Aroma substances are the most crucial criteria for the sensory evaluation of tea quality, and also key attractors influencing consumers to make the decision for purchasing tea. Understanding the aromatic properties of tea infusion during different brewing time is crucial to control the tea aromatic quality. Here, headspace and direct immersion solid-phase microextraction (HS-SPME and DI-SPME), coupled with GC-MS, were employed to investigate the impact of brewing time on the changes of the volatile features of green tea infusion. Esters, aldehydes, alcohols, fatty acids, and alkaloids were the predominant volatile groups from tea infusions. Two to three minutes was identified as the best duration for the tea brewing that can maximize the abundance of aromatic chemicals in the headspace emitted from the tea infusions. The variation of the key aromatic contributors between the tea infusion and the headspace over the infusion tended to equilibrate during the tea brewing process. This study provides a theory-based reference method by analyzing the real-time aromatic characteristics in green tea. The optimal time was determined for aromatic quality control, and the complementary relationship between the volatiles in the headspace and its counterpart, tea infusion, was primarily elucidated.

## 1. Introduction

Green tea, produced by the tea leaves (*Camellia sinensis*), is widely appreciated for its cultural connotations, sensory attributes, and health properties, especially in Asian countries [1]. Aroma, flavor, color, and appearance are primary aspects for evaluating green tea quality. The olfactory attributes of different tea brewing times are related to different components present in tea products [2].

Aroma is perceived as a leading factor defining tea quality that influences the consumer’s selection, acceptance, and digestion of the tea. Volatile organic compounds (VOCs), as the fundamental metabolites for tea (odor and aroma), occur in tea in small quantities, accounting for 0.01% of dry weight, but are of enormous importance to perceptions of quality [3]. More than 200 VOCs have been identified in green tea, including alcohols, aldehydes, acids, ketones, and esters [4]. Constant efforts have been devoted to enhancing the aromatic quality of tea, covering various aspects, from the level of tea plant breeding [5], agronomic practices [6,7], and manufacturing processes [8], to exogenous additions [9]. A previous study discussed tea plantation (‘raw tea’) areas, as well as the accumulation mechanisms of the precursors of aromatic compounds and their associated metabolic pathways. The formation mechanisms of tea aromas mainly comprise six categories, fatty-acids-derived volatiles, terpenoid volatiles, phenylpropanoids/benzenoids, carotenoid derivatives, glycoside hydrolysates, and Maillard reaction products [4,10]. However, the changes of aromatic substances during tea brewing are still unclear.

Following cultivation and processing, the brewing conditions are another pivotal factor that can significantly affect the aroma presenting in the tea infusion that will be directly perceived by consumers. The VOCs from tea infusion consist of numerous volatiles, and are various and highly dynamic. Specifically, some of them appear to have a temporal property, as they occur in a relatively short period, such as during tea brewing, whereas some of them with a persistent trait are relatively stable [11]. The information of the tea aroma quality could potentially be lost, and thereby, would not fully reflect the complex aroma profiles of the tea infusion. Numerous researchers have revealed the impact of different brewing conditions on releasing or the formatting of VOCs in green teas, including the brewing temperature, brewing time, water hardness, leaf size, and the brewing apparatus [12,13]. Studying VOCs of the tea infusion provides a great insight into understanding the functions, compositions, and variation of tea aromas. Traditionally, trained panelists evaluated the tea quality (appearance, aroma, color, and taste) according to the standardized procedure (GB/T23776-2009), and generated a quantitative score for each descriptor. However, it is time consuming, and subject to the influence of external factors. In some cases, there is an urgent demand for the discrimination of a high number of samples with detailed proofing data, which is beyond the sample-scale of the sensory test method [14,15]. Thus, an effective and objective analytical approach is essentially required for the evaluation of the aromatic quality of tea infusion, which can fully cover the manifold temporal changes of volatiles in the tea infusion in a relatively short period. Li et al. [16] applied a highly time-resolved positive photoionization ion mobility spectrometry approach to capture the real-time fingerprinting of the dynamic changes of VOCs, and assessed five types of green tea aromas during brewing (‘tender chestnut-like’, ‘roasted chestnut-like’, ‘fresh’, ‘sweet’, and ‘tender’). However, an in-depth study is still lacking in the kinetics of VOCs released from tea infusion, and the relationship between the volatile profile in tea infusion and the corresponding headspace.

Previously, the study of metabolic profiles of VOCs was mainly dependent on complicated solvent extraction techniques, such as liquid–liquid extraction (LLE) and solid-phase extraction (SPE). These techniques presented some shortages, such as the presence of harmful organic solvents, and expensive devices with a limited lifetime, as well as cross-contamination problems. Solid phase microextraction (SPME), developed by Arthur and Pawliszyn [17], has been regarded as a rapid and simple technique in sample extraction and pre-concentration for further VOCs analysis. Due to the considerable advantages of being solvent-free, and having high sensitivity without producing artifacts, SPME is widely applied to the extraction of VOCs in foods [18]. Extraction is carried out either by direct immersion (DI-SPME), where the fiber is directly immersed in the liquid sample, or headspace SPME (HS-SPME), where the fiber is exposed in the vapor phase above a sample. SPME coupled with the method of real-time sampling of tea infusions can decipher the volatile profiles to some extent. 

In this study, we systematically explored the changes of VOCs, and the potential regulation that governed their formation or releasing between tea leaves, the tea infusion, and the headspace during tea brewing. Sealed syringes were employed to accurately capture the real time VOCs released from green tea infusions into the headspace (HS-SPME). Additionally, direct immersion solid-phase microextraction (DI-SPME) coupled with gas chromatography-mass spectrometry (GC-MS) were employed to investigate the differences and leaching rules of the chemical composition between the tea infusions and the corresponding headspace during different brewing times.

## 2. Materials and Methods

### 2.1. The Preparation of Tea Samples

The young tea shoots (a bud and two expanding leaves, *Camellia sinensis* cv. Huangshanzhong) were collected from an 8-year-old tea plantation located in Qingdao, North China Plain (36°19′ N, 120°23′ E, elevation 54.88 m). The soil properties were described in our previous study [19]. Green tea samples were created by an experienced tea master using the traditional manufacturing processes of withering, heating, rolling, and drying. The results of the sensory test were described in our previous study (S3 sample harvest in late summer) [20]. The tea samples were kept in aluminum foil sachets, and stored in a fridge until further analysis.

### 2.2. SPME Procedure and Sampling Setup

The three-phase 2 cm 50/30 μm Divinylbenzene/Carboxen/Polydimethylsiloxane (DVB/CAR/PDMS, Agilent) fiber and the 1 cm 30 μm Polydimethylsiloxane (PDMS) fiber were employed in this study. C7–C40 saturated alkanes mixture was purchased from Sigma-Aldrich (Castle Hill, Australia). Deionized water was purified through a Milli-Q Biocel system (Millipore, Burlington, MA, USA). 

Three grams of the tea were placed in a 250 mL Erlenmeyer flask, which was used as the tea brewing apparatus, and then, 50 mL of boiling deionized water was added to the flask. Subsequently, the flask was sealed with a PTFE-silicon septum, and the stirring speed was 60 rpm. The green tea was brewed for 1, 2, 3, 4, and 5 min, respectively. Each infusion was performed in triplicate. Both of the HS-SPME and the DI-SPME were conducted to extract the compounds of the tea infusion, respectively. Briefly, for the HS-SPME, a 10 mL syringe was used to remove air from the flask at each time point. It is worth highlighting that the volatile compounds in tea infusions were easily impacted by heat treatment [21]. Therefore, the temperature was set at 40 °C for the extraction. After pre-equilibrate for 30 min at 40 °C in a thermostatic oscillator [13,21], the gas volume in the syringe was adjusted to 5 mL, and then, the SPME fiber was inserted into the syringe through a needle with a septum for 1 h extraction (Supplementary Figure S1). In terms of DI-SPME analysis, 2 mL of the tea infusions was transferred to a 2 mL HPLC amber vial with screw caps PTFE/blue silicone (9 mm), purchased from Agilent. After completing the sampling, all of the samples were immediately put into a thermostatic oscillator at 40 °C for 30 min. Then, the fiber was immersed in the tea infusion for 1 h. After exposure, the fiber was removed and desorbed at 270 °C in the injector port of GC for 10 min.

### 2.3. GC-MS Conditions

An Agilent 7890B gas chromatograph with a capillary column HP-5MS (30 m × 0.25 mm I.D, film thickness 0.25 µm, Agilent, Santa Clara, CA, USA) coupled with a 5977E Mass selective detector (MSD) were used throughout the study. Both the HS-SPME and DI-SPME employed the same GC/MS conditions. Purified helium (99.999%) at a constant flow rate of 1 mL/min was utilized as the carrier gas. The column temperature program was 40 °C for 5 min, and then, increased to 220 °C at a rate of 4 °C/min, and finally increased to 300 °C (at a rate of 50 °C/min), and was held for 5 min. The MS parameters were as follows: ion source temperature was at 230 °C and the spectra was acquired in a range from 35 to 500 atomic mass units (amu) under the electron impact (EI) mode at 70 eV; the transfer line temperature of MSD was at 280 °C, and the MS Quad temperature was at 150 °C, respectively. 

### 2.4. Identification of Compounds

Peak identifications were conducted by searching the mass spectra in the National Institutes of Standards and Technology Mass Spectrometry (NIST MS) library, and comparing their Kovats retention indices (RIs) with the published data. The n-alkane standard at 100 µg/mL (catalog number 49451-U; Castle Hill, NSW, Australia) was injected under the same GC conditions as the external standard.

### 2.5. Statistical Analysis

The data acquisition was performed by MassHunter Acquisition software (version B. 06.00 Agilent Technologies Santa Clara, CA, USA), and data was expressed as the mean ± standard deviation of three replicates. Analyses of variances (ANOVA) and Fisher-HSD at *p* < 0.05 were chosen to investigate statistical differences among means. Furthermore, multivariate statistical techniques, including partial least squares discriminant analysis (PLS-DA) and hierarchical cluster analysis performed by MetaboAnalyst 5.0 (https://www.metaboanalyst.ca/, accessed on 30 October 2021), were used to characterize the green teas based on different brewing times. The relative abundance of the volatile components was obtained by peak area normalization (Log10).

## 3. Results and Discussion

### 3.1. Comparison of Nonpolar and Polar SPME Fibres

Solid phase microextraction (SPME) is a relatively simple method, and is mediated by different fused silica fibers coated with polar or nonpolar adsorbents for the adsorption and desorption of compounds. Though SPME fibers coated with Carboxen/DVB/PDMS are commonly used for polar and nonpolar volatiles, there is a lack of evidence to support whether the PDMS fiber can be more effective in extracting nonpolar volatiles and semi-volatiles. To determine the optimum fiber for efficient extractions, Carboxen/DVB/PDMS fibers and PDMS fibers were used to extract the volatiles of tea infusions under the same extraction conditions of GC-MS optimized by previous references [21,22]. As with the chromatograms shown in Supplementary Figure S2, the Carboxen/DVB/PDMS fiber was found to be more efficient for tea volatile adsorption for both HS-SPME and DI-SPME. In addition, all of the peaks in the chromatogram of the nonpolar fiber were presented in the chromatogram of the polar fiber.

### 3.2. Identification of Compounds in Tea Infusion and the Corresponding Headspace

The alteration of VOCs during tea brewing includes manifold temporal changes involving the aroma’s releasing, maximization, and attenuation. How to create an efficient method to capture the real-time volatile profiles was a challenge. The sealed syringe technique of headspace sampling was developed to obtain the real-time VOCs released from the tea infusion. To mimic the real scenario of tea consumption, and analyze as much of the aroma that would be smelled before tasting, no chemical extraction and no intentional concentration were used in this study. This resulted in the number of compounds being lower than that in previous studies. For HS, a total of 38 VOCs were identified based on the retention time and mass spectrometric data. The identification of VOCs and their characteristics are summarized in Table 1. The number of compounds was lower than that in previous studies with organic solvents, high temperatures, and increasing tea concentrations of sample extractions [23,24]. According to the GC-MS and the related chemical structures, these compounds can be divided into nine types, including six alcohols, seven aldehydes, six esters, six fatty acids, two heterocycles, six hydrocarbons, three ketones, one organosulfide, and one phenol. ANOVA characterized 22/38 volatiles (57.89%) that varied during the tea brewing process (ANOVA, FDR adjusted *p* < 0.05). Regarding DI, a total of 39 compounds in the green tea infusion were identified (Table 2). These compounds comprised nine types, including three alcohols, nine aldehydes, two alkaloids, five esters, seven fatty acids, two heterocycles, five hydrocarbons, three ketones, and three phenols. These constituents are similar to those obtained in previous tea leaves studies [25]. ANOVA characterized 30/39 compounds (76.92%) that varied during the tea brewing process (ANOVA, FDR adjusted *p* < 0.05).

### 3.3. The Effect of Brewing Time on Volatile Profiles

**Figure 1:**
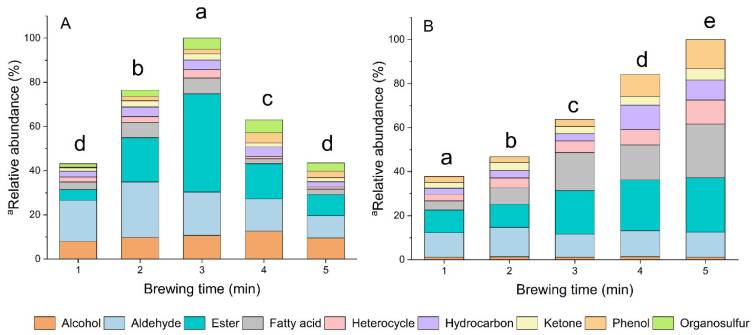
Main contribution groups of compounds shown by the relative abundance. Percentage is the ratio of total peak area of each group to the total peak area of all groups. (**A**) Relative abundance of VOCs in headspace; (**B**) relative abundance of compounds (without alkaloid group) in tea infusion. Different lower-case letters indicate significant difference (*p* < 0.05).

The volatile groups in HS (Figure 1A) and DI (Figure 1B) showed different responses related to the different brewing times. In HS, the total abundance of VOCs increased from 1 min to 3 min, and decreased from 3 min to 5 min, whereas the total volatiles in DI presented a continuously increasing tendency during the whole brewing process. This observation was supported by the differences of the relatively higher molecular weight and more stabilized structures of the compounds present in DI compared to those in HS. In this study, the DI method was employed by obtaining semi-volatile and volatile compounds in tea infusion; however, HS methods were utilized to absorb volatile compounds in the headspace of tea infusion. Compounds gradually dissolved in the tea infusion (DI) during brewing, which explained why an increasing trend was observed in DI. Compounds with smaller molecular weight and higher vapor pressure were easier released into the HS from the tea infusion, but the temperature decreased with a prolonged tea brewing time (Supplementary Figure S3), resulting in less VOCs release after 4 min brewing. It must be emphasized that we only focused on the comparison of the variation tendency between two different phases (HS and DI). Consistent with the previous report, esters, aldehydes, and alcohols appeared to be the main constituents in HS [24]. Unlike the constituents in HS, the main constituents in DI were alkaloids, aldehydes, esters, and fatty acids. Aldehydes and alcohols were considered the dominant volatiles in tea infusions, and were generally characterized as an intense sensory sensation by panelists [21]. Referring to the constituents, the aldehydes and alcohols in our study, most of these had six to ten carbons. Previous investigations of green tea infusions having aldehydes and alcohols with six to ten carbons were perceived as having green, floral, woody, and other pleasant scents [26]. In addition, it was revealed that hot water played a vital role in extracting aldehydic and alcoholic volatiles in teas [27]. The results were in accordance with these published reports, showing an increasing tendency of aldehydic and alcoholic volatiles both in HS and DI under similar conditions. Additionally, alkaloids were the dominating compounds (79.23% on average) in DI (Supplementary Table S1); however, the fact that they could not be detected in HS was attributed to its non-volatile nature. The presence of alkaloids in tea infusions contributed to their flavor and the stimulatory effect of the tea [28]. Fatty acids are well-known precursors of aroma compounds, owing to their contribution to green tea quality. A great number of volatile aroma compounds in teas are the product of the degradation of unsaturated fatty acids [29]. Both higher temperatures and longer brewing times resulted in this variation, which was consistent with the fluctuation of the percentage of total fatty acids in tea infusions. The abundance of esters in HS initially increased from 1 min, reached the highest at 3 min brewing, and gradually decreased with extending brewing time to 5 min. A continuous increasing trend of total esters was observed in DI during the whole brewing time. Esters are mainly originated from the esterification reaction, which converts alcohols and fatty acid into esters. More esters would be dissolved in the tea infusion during brewing. As a reversible reaction, esters undergo hydrolysis into alcohols and fatty acids with an increased brewing time and temperature, and release them into the headspace. 

### 3.4. Differential Analysis and Partial Least Squares-Discriminant Analysis (PLS-DA) of Compounds

**Figure 2:**
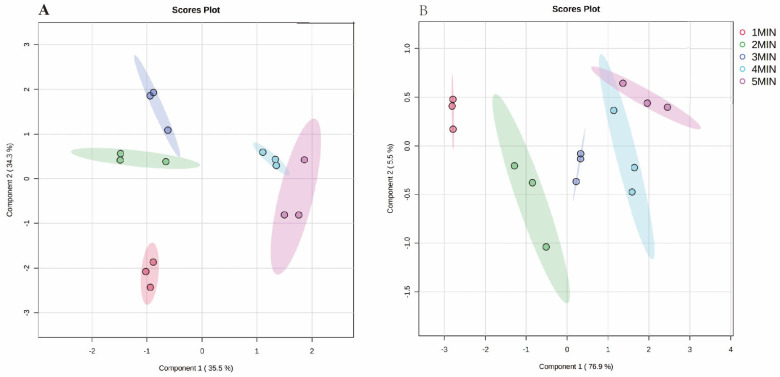
Partial least squares-discriminant analysis (PLS-DA) scores plot for separation of compounds at different brewing times in HS (**A**) and DI (**B**), respectively.

Partial least squares-discriminant analysis (PLS-DA), as a supervised classification method, can overcome the shortcomings of PCA, enhance the differences between groups, and provide an indicator that is responsible for separation [30]. In this study, PLS-DA was conducted to compare the volatile profiles of green teas under five different brewing times (Figure 2). The volatile components identified were defined as the X variables, and different brewing times were assigned as the Y variables. For HS (Figure 2A), the principal components (PC) 1 and 2 explained the 35.5% and 34.3% of variation of the data, respectively. The most important volatile compounds contributing negatively to PC1 were linalool oxide, 1-eicosane, benzeneacetaldehyde, octanoic acid, and tetradecanoic acid, which have a floral character. 2,4-di-tert-butylphenol was the most important positive contributor to PC1. The compounds that mostly contributed to PC2 were phthalic acid, butyl hex-3-yl ester, octanoic acid, and isophytol. For DI (Figure 2B), PC 1 explained 76.9% of the variation. The most important volatile compounds contributing positively to PC1 were 1,2,3-benzenetriol, n-hexadecanoic acid, and 9-hexadecenoic acid. PC 2 explained the 5.5% of variation and limonene, theobromine, n-hexadecanoic acid, and 1,2,3-benzenetriol. The score plots emphasized the separation between samples among the five groups. This indicated that the brewing time exerted a considerable role in altering the composition of the compounds in tea infusions. Based on the PC1, the chemical groups in HS can be divided into two groups: group 1 (1, 2, and 3 min); and group 2 (4 and 5 min). In the DI, despite the slight overlap between 4 min and 5 min, the other chemical groups could separate well. The value of R2 and Q2 (0.9894 and 0.8977 in HS, respectively; 0.9926 and 0.9578 in DI, respectively) proved that the PLS-DA model had a good fitness and validity.

### 3.5. Key Individual Compounds in Tea Infusion and the Corresponding Headspace

Variable importance in projection (VIP) reflects the importance of variables in PLS-DA classification. The value of a VIP score larger than 1.0 is of primary importance in selecting relevant variables [31]. 

**Figure 3:**
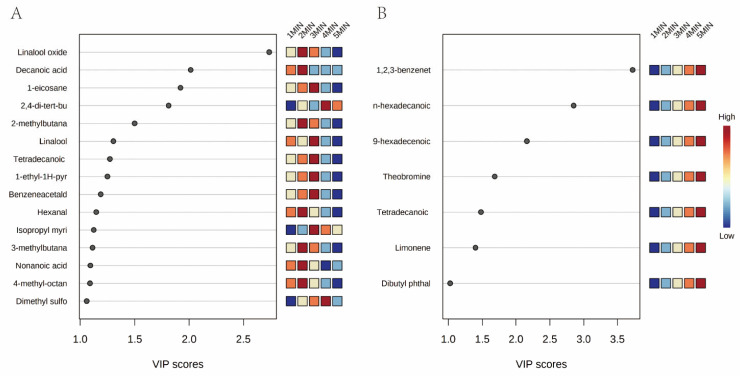
Key individual compounds in headspace (**A**) and the corresponding tea infusion (**B**) based on the VIP scores (VIP > 1). Colored boxes on the right indicate the relative concentrations of the compounds under different brewing times.

In the HS, the following 15 compounds with VIP > 1.0 (Figure 3A and Table 3) were identified: linalool oxide; n-decanoic acid; 1-eicosane; 2,4-di-tert-butylphenol; 2-methylbutanal; linalool; tetradecanoic acid; 1-ethyl-1H-pyrrole; benzeneacetaldehyde; hexanal; isopropyl myristate; 3-methylbutanal; nonanoic acid 4-methyl-octane; and dimethyl sulfone. All of these displayed an initial increasing, and then decreasing, tendency. Most of the compounds showed the highest concentration at the two- or three-minute point, except for dimethyl sulfide and 2,4-di-tert-butylphenol. Volatile terpenoids are an important class of aroma-active compounds that are responsible for the flavor and fragrance of food. Linalool and linalool oxide are terpene alcohols, which have floral and lavender aromas [32]. Linalool oxide appeared to be the dominant differential compound (VIP value 2.700) present in the headspace during the green tea brewing process. Linalool was considered as the product of the carotenoid degradation, and the primary oxidation of phytoene, phytofluene, and lycopene [2]. Baba et al. [33] reported that linalool was crucial to the characteristic green tea aroma. Free linalool was reported to be released from glycoside through heating [34], which could explain the increasing concentration of the linalool, as shown in Table 2. Racemic linalool oxides included four types, named linalool oxide A-D [35]. Instead of coming from the oxidization of linalool, linalool oxides presented as the glycoside forms in tea leaves [36]. In this study, the different forms of linalool oxide could not be identified because of the limited current lab capacity and budget. Isopropyl myristate is an odorless compound [37]. Even though many studies have detected it in teas, there was not much evidence showing the variation mediated by the brewing times. Benzeneacetaldehyde, hexanal, 2-methylbutanal, and 3-methylbutanal are aldehydes and well-known flavor compounds in green tea. Benzeneacetaldehyde (synonym: phenylacetaldehyde) mainly exhibits sweet and honey flavors. The product is synthesized from phenylalanine through oxidative degradation or enzymatic oxidation, and the high temperature treatment could increase the concentration of benzeneacetaldehyde [38]. Hexanal is produced through the metabolism of fatty acids, and contributes the grassy, tallow, and fat characteristics to the tea aroma [39]. Ho et al. [2] found that hexanal was sensitive to high temperatures, and could easily be degraded, which was in line with our result that hexanal substantially increased in the first two minutes, and decreased in the following three minutes. 2-methylbutanal and 3-methylbutanal contributed the cocoa, almond, and malt flavors to the tea [40]. It was reported 2-methylbutanal and 3-methylbutanal presented higher odor-activity values than the other compounds, playing a key role in the aroma of oolong tea [21]. Decanoic acid, nonanoic acid, and tetradecanoic acid (myristic acid) are straight saturated fatty acids. Decanoic acid and nonanoic acid were shown as the main constituents of the acidic fractions of tea volatiles [23,41], whereas tetradecanoic acid is a mild and sweet-smelling aromatic acid [42]. 1-Eicosane (Icosane) and 4-methyl-octane are alkane hydrocarbons that can promote the formation of a unique flavor in tea [43,44]. 1-ethyl-1h-pyrrole provides burned and sweet odors, which could not be detected in fresh leaves, as it was formed through the Maillard reaction during the manufacturing process [45]. 2,4-Di-tert-butylphenol is an alkylated phenol compound. Zhao et al. [46] summarized that at least 169 species of organisms can produce 2,4-di-tert-butylphenol, including bacteria, fungi, diatom, monocots, and animals. However, there is no report describing its aroma or roles in tea aroma, even though it has been detected in tea [47]. Volatile sulfur compounds provide subtle flavor characteristics or background sensory nuances to many foods. The sulfur compounds are inclined to present a pleasant flavor at a low concentration, whereas at a high concentration, the aroma was perceived as an off-flavor [48]. Taken together, the majority of the key individual VOCs were significant contributors to the tea aroma, and the brewing time performed a vital role in their releasing from the tea infusion. 

Regarding DI, a total of 7 of 39 compounds appeared to possess their VIP above 1 (Figure 3B and Table 3), including 1,2,3-benzenetriol, n-hexadecanoic acid, 9-hexadecenoic acid, theobromine, tetradecanoic acid, limonene, and dibutyl phthalate. Compared with HS, most of them were relatively larger molecules, with less volatility that does not vaporize readily into the HS. This resulted in markedly different characteristic compounds in the tea infusions (DI). Fatty acids, such as n-hexadecanoic acid, 9-hexadecenoic acid, and tetradecanoic acid, are well-known precursors of aroma compounds, including hexanal, (E)-2-hexanol, and methyl jasmonate [2]. Limonene has turpentine and lemon-like aromas, and the unique enantiomer ratio of limonenes can be used to identify one of the Longjing teas [39]. Dibutyl phthalate (DBP) is one of the most abundant phthalates in the environment, owing to its wide usage as a plasticizer and additive in plastics, cosmetics, paints, and pesticides [49]. In some countries, tea is usually commercialized in tea filter bags made of materials such as paper and a variety of plastics. These materials may contain phthalates that can be released into solutions of teas at high temperature. Yamanishi et al. [50] reported that dibutyl phthalate was identified as a constituent in green tea flavor, and the main component in the eluates of the Dianhong tea extract [51]. However, because of its carcinogenic and estrogenic impact on human health, the US Environmental Protection Agency and the European Food Safety Authority proclaim that phthalate esters have been classified as reproductive toxicants, and can only be used with specific authorization [52]. The specific migration limits and the tolerable daily intakes (TDIs) for certain phthalates have been established by the European Commission and European Food Safety Authority (EFSA). Though many studies showed that phthalate esters in teas would not cause high risk, taking into account the tolerable daily intakes, more research effort is needed to clarify the toxicity level of more pollutants, and to remove them efficiently [49,53].

**Table 3:** Flavor description of the key compounds in headspace and the corresponding tea infusion.

Chemical Groups		^a^ No.	Compounds	^b^ Flavour Description
HS	Terpene alcohol	2	Linalool oxide	Floral [32]
		3	Linalool	Floral, lavender [34]
		6	Isopropyl myristate	Odorless [36]
	Aldehyde	8	2-Methylbutanal	Cocoa, almond [37]
		11	Benzeneacetaldehyde	Vanilla-like [38]
		9	Hexanal	Grass, tallow, fat [39]
		7	3-Methylbutanal	Malt [37]
	Fatty acid	21	Decanoic acid	Soapy, waxy, fruity [23]
		23	Tetradecanoic acid	Faint oily [42]
		20	Nonanoic acid	Sweet, vanilla [41]
	Hydrocarbon	28	1-Eicosane	Hydrocarbon-like [43]
		29	4-Methyloctane	Alkane [44]
	Heterocycle	26	1-Ethyl-1H-pyrrole	Burnt and sweet [45]
	Alkylated phenol	38	2,4-Di-tert-butylphenol	NA
	Sulfur	37	Dimethyl sulfone	Laver-like [48]
DI	Phenol	39	1,2,3-Benzenetriol	Astringent and bitter taste [54]
	Fatty acid	23	n-Hexadecanoic acid	Precursors of aroma compounds [2]
		26	9-Hexadecenoic acid	
		20	Tetradecanoic acid	
	Alkaloid	13	Theobromine	Bitter taste [55]
	Hydrocarbon	29	Limonene	Turpentine- and lemon-like aromas [39]
	Ester	18	Dibutyl phthalate	Component of green flavor [50]

^a^ Numbers shown in Table 1 and Table 2. ^b^ Aroma description referred from the literature.

### 3.6. Elucidation of the Relationship between VOCs in Tea Infusion and the Correspounding Headspace

**Figure 4:**
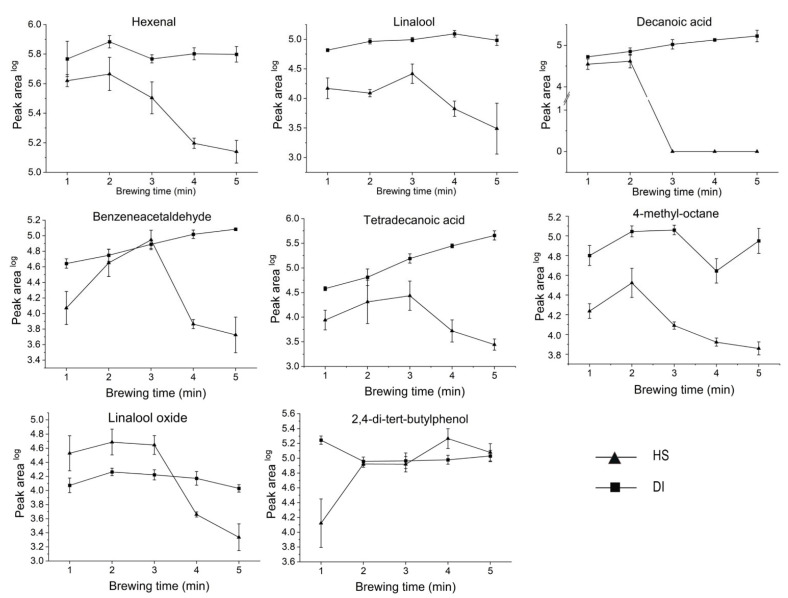
Dynamic changes of common compounds (peak area) in headspace and tea infusion.

Based on the pathways of the tea aroma formation, two types of reactions could be summarized as the main contributors, enzymatic reactions (occurring in the preharvest tea plant growth and postharvest processing), and the thermophysical and chemical reactions (occurring in the partial postharvest processing, storage, and consumption stages) [56]. Tea brewing can be regarded as the process of substances dissolving, and the flavor formation in the tea infusion, which conforms to the hypothesis of ternary phase diagrams. The phase equilibrium for the tea–water system comprises three components: water, tea leaves (soluble tea solids and insoluble tea solids), and headspace. In other words, the system tends to equilibrate during the tea brewing process, including the VOCs released from the tea leaves into the tea infusion, and the VOCs released from the tea infusion into the headspace through thermophysical and chemical reactions. It has been reported that the transfer of VOCs from the interior of tea leaves across the leaf/water interface was the equilibrium-rate-determining step [57]. Furthermore, the properties and the percentage of the compounds in tea leaves, especially soluble substances, as well as the brewing conditions, played crucial roles in this step. As shown in the Supplementary Figure S3, a high-temperature water in the initial time (1 min to 3 min) could lead to a higher rate of VOC transfer between the tea leaves and the water, and the effect tended to be weaker as the temperature gradually decreased from 92.00 to 62.37 °C. This phenomenon was supported by the variation of the water extract during the continuously increasing time from 1 min to 4 min, and then, levelling it out at 5 min. In other words, it means the motion of the volatiles was reduced by lowering the temperature, strongly affecting their release, maximization, and attenuation. However, for DI, it was observed, qualitatively, that the increase in concentration of its VOCs coincided more with increased brewing time. With regard to the heterogeneous nature of tea leaves, Long VD [58] divided tea solubles into three classes: effectively instantaneous solubles, rapid solubles, and slower solubles. The instantaneous and rapid soluble substances must be immediately accessible to water. The slower dissolving components, due to their higher molecular weight, diffuse more slowly from inside to outside, or through the leaf matrix to the water. The research demonstrated that the concentration of the extracted VOCs in the tea infusion displayed a considerable increase during the initial brewing time (1 to 5 min). The rate-determining step for diffusion was mainly determined by the rapid soluble compounds and slower soluble compounds. As indicated in heatmaps (Supplementary Figure S4), the majority of VOCs in the headspace appeared to first increase, and then, decrease with extended brewing times, whereas the counterparts in the tea infusions roughly exhibited a continuously increasing trend. The possible explanation for this is that compounds continuously dissolve in the tea infusions with extended brewing time, resulting in increasing concentrations. The alteration of concentrations of different compounds in HS were the results of integrative actions of the decreasing temperature and the increased brewing time. As a result, in comparation of the results of HS and DI, 17 of 38 volatiles in HS samples can be detected in DI samples, including eight characteristic VOCs in HS samples (Supplementary Figure S5). The variation of eight common compounds (VIP > 1 in HS, hexenal, linalool, decanoic acid, benzeneacetaldehyde, tetradecanoic acids, 4-methy-octane, linalool oxides, and 2,4-di-tert-butylphenol) in both HS and DI presented a complementary tendency (Figure 4). The decreasing concentration of VOCs in HS caused the increased concentration of volatiles in DI, except for 4-methyoctane and 2,4-di-tert-butylphenol. These two exceptions could occur because the variation of 4-methyoctane and 2,4-di-tert-butylphenol may have been oxidized or degraded to some other undetected macromolecules.

## 4. Conclusions

In summary, our study revealed the changes of the volatiles in tea infusion and the corresponding headspace. The performance of volatiles in the headspace and the tea infusions was the result of the interaction between the dissolution of compounds in tea leaves; the temperature variation of the tea infusion; and the physical and chemical transference among the leaves, the water, and the headspace to reach an equilibrium. Chemical groups in HS and DI showed various patterns when subjected to different brewing times. Additionally, the variation of the common VOCs in the headspace and the tea infusions displayed a complementary trend. The contribution of individual VOCs in HS and DI was discussed to provide a theoretical reference on the best time selection for the aromatic quality assessment. Two to three minutes were identified as the best time points for the tea brewing to release the maximum amount of aromatic chemicals from the tea infusions into the headspace. To the best of our knowledge, this is the first comprehensive study describing VOC characterization in the headspace and the corresponding tea infusion, and the relationship of VOCs in these two phases (HS and DI). The relationship between these two phases requires further investigation.

## Figures and Tables

**Table 1:** Volatile compounds detected by HS-SPME-GC-MS during different brewing times.

No.	Chemical Groups	Compounds	RT ^a^	RI ^b^	RI (cal) ^c^	Individual Peak Area ^d^ under Different Brewing Time (min)				
						1	2	3	4	5
1	Alcohol (6)	2-Ethyl-1-hexanol	19.20	1030	1101	4.77 ± 0.15 a	4.75 ± 0.04 a	4.76 ± 0.06 a	4.85 ± 0.01 a	4.64 ± 0.23 a
2		Linalool oxide *	20.44	1077	1058	4.53 ± 0.25 a	4.69 ± 0.18 a	4.65 ± 0.13 a	3.66 ± 0.04 b	3.34 ± 0.19 b
3		Linalool *	22.12	1146	1099	4.17 ± 0.17 a	4.09 ± 0.06 ab	4.42 ± 0.16 a	3.83 ± 0.13 ab	3.49 ± 0.43 b
4		(E)-2-Nonen-1-ol	22.27	1176	1104	5.19 ± 0.23 a	5.31 ± 0.17 a	5.39 ± 0.09 a	5.48 ± 0.04 a	5.41 ± 0.11 a
5		(E)-2-Decen-1-ol *	24.81	1257	1188	4.58 ± 0.09 b	4.72 ± 0.10 ab	4.51 ± 0.08 b	5.07 ± 0.16 a	4.81 ± 0.26 ab
6		Isophytol *	46.02	1960	1949	n.d.	3.80 ± 0.15 b	4.28 ± 0.26 a	n.d.	n.d.
7	Aldehyde (7)	3-Methylbutanal *	3.92	633	631	4.88 ± 0.09 ab	5.17 ± 0.15 a	5.07 ± 0.05 a	4.57 ± 0.05 bc	4.49 ± 0.27 c
8		2-Methylbutanal *	4.15	643	645	4.74 ± 0.05 bc	5.14 ± 0.21 a	5.05 ± 0.17 ab	4.57 ± 0.07 c	4.09 ± 0.09 d
9		Hexanal *	9.00	800	798	5.62 ± 0.04 ab	5.67 ± 0.11 a	5.50 ± 0.11 ab	5.20 ± 0.03 ab	5.14 ± 0.08 b
10		Heptanal	13.26	890	892	4.36 ± 0.15 a	4.39 ± 0.05 a	4.21 ± 0.17 a	4.29 ± 0.22 a	4.34 ± 0.13 a
11		Benzeneacetaldehyde *	19.91	1044	1162	4.07 ± 0.21 b	4.65 ± 0.18 a	4.95 ± 0.13 a	3.86 ± 0.06 b	3.73 ± 0.23 b
12		Decanal	26.17	1206	1231	5.04 ± 0.13 a	5.11 ± 0.17 a	5.07 ± 0.05 a	5.48 ± 0.19 a	5.02 ± 0.56 a
13		Dodecanal	33.16	1409	1471	4.26 ± 0.09 a	4.01 ± 0.01 a	4.00 ± 0.15 a	4.02 ± 0.11 a	4.04 ± 0.15 a
14	Ester (6)	3-Methyl-1-butanol acetate *	12.63	876	878	4.43 ± 0.11 b	4.84 ± 0.08 a	4.72 ± 0.03 ab	4.08 ± 0.21 c	4.46 ± 0.08 d
15		Pentanoic acid, 2,2,4-trimethyl-3-carboxyisopropyl	38.99	1581	1676	3.87 ± 0.07 a	3.90 ± 0.37 a	3.85 ± 0.14 a	3.90 ± 0.35 a	3.87 ± 0.16 a
16		2,2,4-Trimethyl-1,3-pentanediol diisobutyrate	39.08	1588	1679	4.45 ± 0.40 a	4.60 ± 0.20 a	4.75 ± 0.40 a	4.93 ± 0.02 a	4.70 ± 0.28 a
17		Isopropyl myristate	45.20	1827	1916	4.72 ± 0.34 a	4.77 ± 0.62 a	5.33 ± 0.80 a	5.28 ± 0.12 a	5.17 ± 0.11 a
18		Dibutyl phthalate *	46.47	1965	1967	3.61 ± 0.32 c	4.39 ± 0.26 ab	4.61 ± 0.15 a	4.33 ± 0.12 ab	3.90 ± 0.10 bc
19		Phthalic acid, butyl hex-3-yl ester *	48.76	2064	2065	4.30 ± 0.83 b	5.72 ± 0.15 ab	6.04 ± 0.26 a	5.5 ± 0.16 ab	4.58 ± 0.91 ab
20	Fatty acid (6)	Nonanoic acid *	28.22	1273	1304	4.78 ± 0.07 a	4.83 ± 0.05 a	4.44 ± 0.08 b	4.33 ± 0.13 b	4.35 ± 0.07 b
21		Decanoic acid *	31.60	1373	1416	4.55 ± 0.12 a	4.62 ± 0.16 a	n.d.	n.d.	n.d.
22		3,4-Dimethoxycinnamic acid *	45.71	1926	1934	3.79 ± 0.21 b	4.13 ± 0.20 ab	4.43 ± 0.06 a	4.15 ± 0.25 ab	3.96 ± 0.21 ab
23		Tetradecanoic acid *	43.38	1768	1843	3.94 ± 0.20 ab	4.31 ± 0.44 a	4.44 ± 0.30 a	3.72 ± 0.22 ab	3.44 ± 0.11 a
24		Hexanoic acid *	16.98	990	976	4.30 ± 0.08 b	4.67 ± 0.14 a	4.60 ± 0.07 a	4.53 ± 0.15 ab	4.76 ± 0.09 a
25		Octanoic acid *	24.72	1180	1184	3.65 ± 0.10 c	4.90 ± 0.06 a	5.22 ± 0.03 a	4.08 ± 0.19 b	3.69 ± 0.17 c
26	Heterocycles (2)	1-Ethyl-1H-pyrrole *	9.74	821	814	4.78 ± 0.37 ab	4.93 ± 0.23 ab	5.08 ± 0.14 a	4.38 ± 0.19 b	4.28 ± 0.28 b
27		3-Acetyl-2,5-dimethylfuran	22.70	1099	1118	4.17 ± 0.31 a	4.18 ± 0.18 a	4.40 ± 0.25 a	4.26 ± 0.28 a	4.31 ± 0.25 a
28	Hydrocarbon (6)	1-Eicosane *	46.60	1993	1973	3.89 ± 0.18 b	4.09 ± 0.03 ab	4.44 ± 0.38 a	3.71 ± 0.20 b	n.d.
29		4-Methyloctane *	11.93	863	863	4.24 ± 0.07 b	4.52 ± 0.15 a	4.09 ± 0.04 bc	3.92 ± 0.04 cd	3.86 ± 0.07 d
30		2-Methyl-decane	20.63	1064	1063	3.90 ± 0.14 a	4.25 ± 0.43 a	4.53 ± 0.08 a	4.62 ± 0.38 a	4.01 ± 0.39 a
31		2-Methylundecane *	22.18	1164	1101	3.49 ± 0.12 b	4.33 ± 0.51 a	4.21 ± 0.13 a	3.96 ± 0.07 ab	3.83 ± 0.20 ab
32		2,3,5,8-Tetramethyldecane	28.80	1318	1323	4.74 ± 0.20 a	4.69 ± 0.26 a	4.79 ± 0.08 a	4.92 ± 0.18 a	4.71 ± 0.27 a
33		Heptadecane	41.82	1700	1681	4.05 ± 0.16 ab	4.15 ± 0.24 ab	4.25 ± 0.02 a	3.93 ± 0.25 ab	3.71 ± 0.08 b
34	Ketone (3)	6-Methyl-5-hepten-2-one	17.48	986	987	4.35 ± 0.22 a	4.79 ± 0.04 a	4.61 ± 0.02 a	4.29 ± 0.28 a	4.17 ± 0.53 a
35		(E)-6,10-Dimethyl-5,9-undecadien-2-one *	34.61	1453	1521	4.01 ± 0.03 ab	4.32 ± 0.30 a	4.09 ± 0.17 ab	3.82 ± 0.16 b	4.22 ± 0.14 ab
36		2,5-Di-tert-butyl-1,4-benzoqui-none	35.24	1466	1543	4.37 ± 0.29 a	4.37 ± 0.29 a	4.77 ± 0.19 a	4.51 ± 0.35 a	4.50 ± 0.29 a
37	Organosulfur (1)	Dimethyl sulfone	14.49	922	919	4.53 ± 0.59 a	5.04 ± 0.15 a	5.29 ± 0.14 a	5.38 ± 0.06 a	5.03 ± 0.43 a
38	Phenol (1)	2,4-Di-tert-butylphenol *	36.43	1519	1584	4.12 ± 0.33 b	4.92 ± 0.04 a	4.92 ± 0.10 a	5.27 ± 0.13 a	5.08 ± 0.12 a

^a^ RT, retention time. ^b^ RI, retention index based on the NIST database. ^c^ RI(cal), retention index based on alkane series. ^d^ Peak areas were normalized by generalized logarithm transformation (Log10). * Statistically significant with *p*-value < 0.05. Different lower-case letters indicate significant difference (*p* < 0.05).

**Table 2:** Compounds detected by DI-SPME-GC-MS during different brewing times.

No.	Chemical Groups	Compounds	RT ^a^	RI ^b^	RI (cal) ^c^	Individual Peak Area ^d^ under Different Brewing Time (min)				
						1	2	3	4	5
1	Alcohol (3)	Linalool oxide *	19.96	1064	1058	4.07 ± 0.10 b	4.26 ± 0.05 a	4.22 ± 0.07 a	4.17 ± 0.10 a	4.03 ± 0.05 a
2		Linalool *	21.71	1099	1100	4.82 ± 0.02 a	4.97 ± 0.04 ab	4.99 ± 0.04 b	5.09 ± 0.06 b	4.98 ± 0.09 b
3		Isophytol *	46.43	1961	1894	4.81 ± 0.09 a	4.71 ± 0.05 a	4.54 ± 0.11 a	4.48 ± 0.15 a	4.56 ± 0.11 a
4	Aldehyde (9)	β-Cyclocitral	26.41	1220	1226	4.43 ± 0.12 a	4.47 ± 0.04 a	4.43 ± 0.10 a	4.48 ± 0.05 a	4.50 ± 0.03 a
5		Undecanal	29.35	1307	1309	4.48 ± 0.12 a	4.48 ± 0.09 a	4.52 ± 0.13 a	4.60 ± 0.05 a	4.58 ± 0.07 a
6		Nonanal *	21.89	1104	1105	5.26 ± 0.04 a	5.11 ± 0.08 abc	5.02 ± 0.04 c	5.18 ± 0.04 ab	5.08 ± 0.07 bc
7		Hexanal	9.01	800	805	5.77 ± 0.12 a	5.88 ± 0.04 a	5.77 ± 0.03 a	5.80 ± 0.04 a	5.80 ± 0.05 a
8		Heptanal *	13.34	901	902	4.44 ± 0.02 ab	4.55 ± 0.13 a	4.32 ± 0.04 b	4.45 ± 0.03 ab	4.56 ± 0.02 a
9		Dodecanal	32.73	1409	1410	4.23 ± 0.14 a	4.49 ± 0.18 a	4.39 ± 0.20 a	4.53 ± 0.14 a	4.40 ± 0.02 a
10		Decanal *	25.74	1206	1206	4.97 ± 0.03 b	5.05 ± 0.06 ab	5.11 ± 0.08 ab	5.20 ± 0.06 a	5.07 ± 0.07 ab
11		Benzeneacetaldehyde *	19.47	1045	1046	4.64 ± 0.06 d	4.75 ± 0.08 cd	4.89 ± 0.06 bc	5.02 ± 0.05 ab	5.08 ± 0.02 a
12		1-Ethyl-1h-pyrrole-2-carboxaldehyde *	19.69	1016	1050	5.58 ± 0.04 ab	5.70 ± 0.05 a	5.49 ± 0.04 b	5.49 ± 0.06 b	5.49 ± 0.03 b
13	Alkaloid (2)	Theobromine *	46.06	1910	1880	5.28 ± 0.24 b	5.32 ± 0.29 b	6.04 ± 0.11 a	6.20 ± 0.17 a	6.42 ± 0.15 a
14		Caffeine	45.67	1835	1856	7.93 ± 0.10 a	8.05 ± 0.08 a	8.03 ± 0.04 a	8.07 ± 0.08 a	7.97 ± 0.06 a
15	Ester (5)	Methyl jasmonate *	40.08	1638	1655	4.99 ± 0.10 c	5.10 ± 0.09 bc	5.13 ± 0.10 bc	5.25 ± 0.05 ab	5.47 ± 0.08 a
16		Hexanoic acid, octyl ester *	38.60	1571	1601	5.80 ± 0.12 a	5.65 ± 0.48 a	6.14 ± 0.63 a	6.59 ± 0.06 a	7.23 ± 0.15 a
17		Dihydroactinidiolide *	36.81	1532	1538	5.57 ± 0.05 b	5.63 ± 0.05 ab	5.66 ± 0.05 ab	5.75 ± 0.06 a	5.78 ± 0.08 a
18		Dibutyl phthalate *	48.29	1965	1968	5.09 ± 0.16 b	5.10 ± 0.14 b	5.59 ± 0.07 a	5.73 ± 0.05 a	5.74 ± 0.06 a
19		3-Methyl-1-butanol acetate	12.87	876	892	4.66 ± 0.08 a	4.54 ± 0.07 a	4.61 ± 0.02 a	4.69 ± 0.08 a	4.68 ± 0.13 a
20	Fatty acid (7)	Tetradecanoic acid *	42.90	1768	1757	4.58 ± 0.04 c	4.81 ± 0.17 c	5.19 ± 0.09 b	5.45 ± 0.04 ab	5.66 ± 0.09 a
21		Oleic acid *	50.70	2141	2103	4.41 ± 0.14 c	4.56 ± 0.11 bc	4.55 ± 0.06 bc	4.78 ± 0.10 ab	4.84 ± 0.08 a
22		Octanoic acid *	24.99	1180	1187	4.83 ± 0.07 bc	5.08 ± 0.06 ab	5.19 ± 0.15 a	5.12 ± 0.13 ab	4.72 ± 0.18 c
23		n-Hexadecanoic acid *	48.19	1968	1961	3.79 ± 0.24 d	5.38 ± 0.28 c	5.55 ± 0.09 bc	6.03 ± 0.08 sb	6.15 ± 0.26 s
24		Decanoic acid *	31.53	1361	1370	4.72 ± 0.03 c	4.85 ± 0.09 bc	5.02 ± 0.11 ab	5.13 ± 0.03 a	5.22 ± 0.14 a
25		Acetic acid *	4.83	610	606	5.52 ± 0.02 c	5.52 ± 0.03 c	6.10 ± 0.02 a	5.10 ± 0.06 d	5.80 ± 0.06 b
26		9-Hexadecenoic acid *	47.64	1942	1942	n.d.	4.64 ± 0.09 c	4.88 ± 0.35 bc	5.24 ± 0.17 ab	5.61 ± 0.12 a
27	Heterocycles (2)	Indole *	28.96	1295	1296	5.45 ± 0.05 e	5.60 ± 0.05 d	5.73 ± 0.04 c	5.88 ± 0.03 b	6.08 ± 0.05 a
28		1-Ethyl-1h-pyrrole	9.41	815	814	5.03 ± 0.06 a	5.17 ± 0.21 a	5.05 ± 0.25 a	5.05 ± 0.08 a	5.21 ± 0.11 a
29	Hydrocarbon (5)	Limonene *	18.89	1017	1032	4.98 ± 0.15 b	5.11 ± 0.22 ab	5.14 ± 0.26 ab	5.81 ± 0.62 ab	5.95 ± 0.07 a
30		Heptadecane	41.32	1700	1698	4.35 ± 0.29 a	4.38 ± 0.18 a	4.45 ± 0.05 a	4.53 ± 0.08 a	4.59 ± 0.12 a
31		4-Methyl-octane *	11.21	863	857	4.80 ± 0.10 ab	5.05 ± 0.06 a	5.06 ± 0.05 a	4.64 ± 0.12 b	4.95 ± 0.13 a
32		2-Methyl-1-propenyl-benzene	21.49	1072	1095	4.65 ± 0.08 a	4.78 ± 0.08 a	4.66 ± 0.11 a	5.07 ± 0.53 a	4.74 ± 0.23 a
33		1-Phenyl-1-butene *	21.20	1098	1087	5.00 ± 0.03 a	4.92 ± 0.07 ab	4.82 ± 0.10 abc	4.74 ± 0.06 bc	4.69 ± 0.09 c
34	Ketone (3)	β-Ionone *	35.33	1491	1493	5.03 ± 0.11 b	5.27 ± 0.07 a	5.24 ± 0.05 a	5.26 ± 0.05 a	5.39 ± 0.08 a
35		Jasmone *	32.57	1394	1404	5.16 ± 0.13 b	5.31 ± 0.06 ab	5.23 ± 0.05 b	5.36 ± 0.08 ab	5.46 ± 0.05 a
36		Cuminone *	36.32	1585	1525	4.88 ± 0.07 b	4.84 ± 0.06 b	4.86 ± 0.08 b	4.88 ± 0.05 b	5.05 ± 0.06 a
37	Phenols (3)	Menthol *	24.58	1169	1176	5.22 ± 0.03 ab	5.30 ± 0.03 a	5.14 ± 0.04 b	4.87 ± 0.06 c	4.85 ± 0.03 c
38		2,4-Di-tert-butylphenol *	35.98	1519	1513	5.25 ± 0.06 a	4.96 ± 0.06 b	4.97 ± 0.10 b	4.98 ± 0.06 b	5.03 ± 0.08 b
39		1,2,3-Benzenetriol (pyrogallol) *	31.60	1386	1376	n.d.	4.41 ± 0.59 b	5.23 ± 0.08 ab	5.88 ± 0.53 a	5.99 ± 0.59 a

^a^ RT, retention time. ^b^ RI, retention index based on the NIST database. ^c^ RI(cal), retention index based on alkane series. ^d^ Peak areas were normalized by generalized logarithm transformation (Log10). * Statistically significant with *p* < 0.05. Different lower-case letters indicate significant difference (*p* < 0.05).

## Data Availability

The data presented in this study are available within the article.

## Supplementary Materials

The following are available online at https://www.mdpi.com/article/10.3390/foods11050684/s1. Table S1. Percentage of VOC groups in DI; Figure S1, Schematic diagram of the research process; Figure S2. Chromatograms obtained after separation of compounds using 50/30 µm Carboxen/DVB/PDMS fiber and PDMS fiber. A, the chromatogram of HS using Carboxen/DVB/PDMS fiber, PDMS fiber, respectively; B, the chromatogram of DI using 30 µm Carboxen/DVB/PDMS fiber, PDMS fiber, respectively; Figure S3. The variation of temperature and water during the tea brewing; Figure S4. The dynamic changes of VOCs during the different brewing time shown by heatmaps; Figure S5. Common compounds in HS and DI.

## Abbreviations

VOCs, volatile organic compounds; SPME, solid phase microextraction; HS, headspace; DI, direct immersion; GC-MS, gas chromatography-mass spectrometry; NIST, national institute of standards and technology; PLS-DA, partial least squares-discriminant analysis; VIP, variable importance in the projection; LLE, liquid–liquid extraction; SPE, solid-phase extraction; MSD, mass-selective detection; DBP, dibutyl phthalate.
