# Supplementary figures and images for: Profiling Real-Time Aroma from Green Tea Infusion during Brewing
Source: Foods. 2022 Feb 25;11(5):684. doi: 10.3390/foods11050684 (PMC8909371; doi:10.3390/foods11050684)

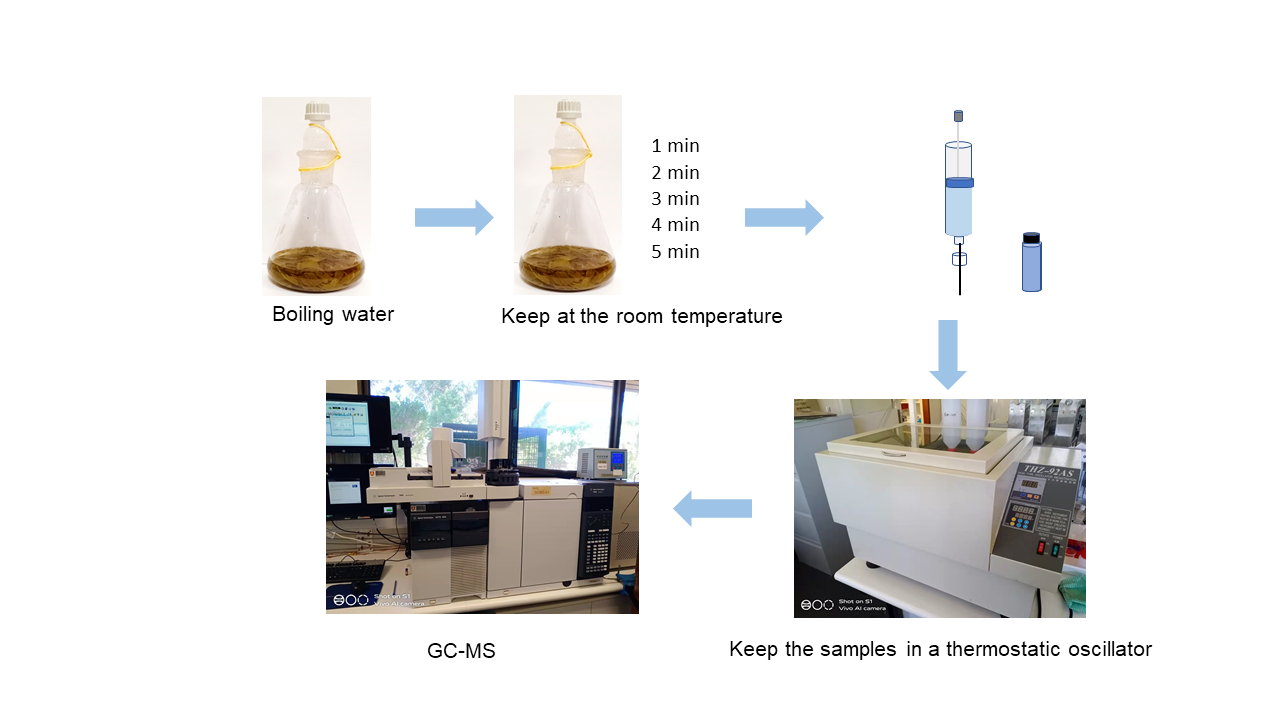

Supplement: Supplementary file 1 [file foods-11-00684-s001.zip › Supplementary documents/Figure S1 Schematic diagram of the research process.png]

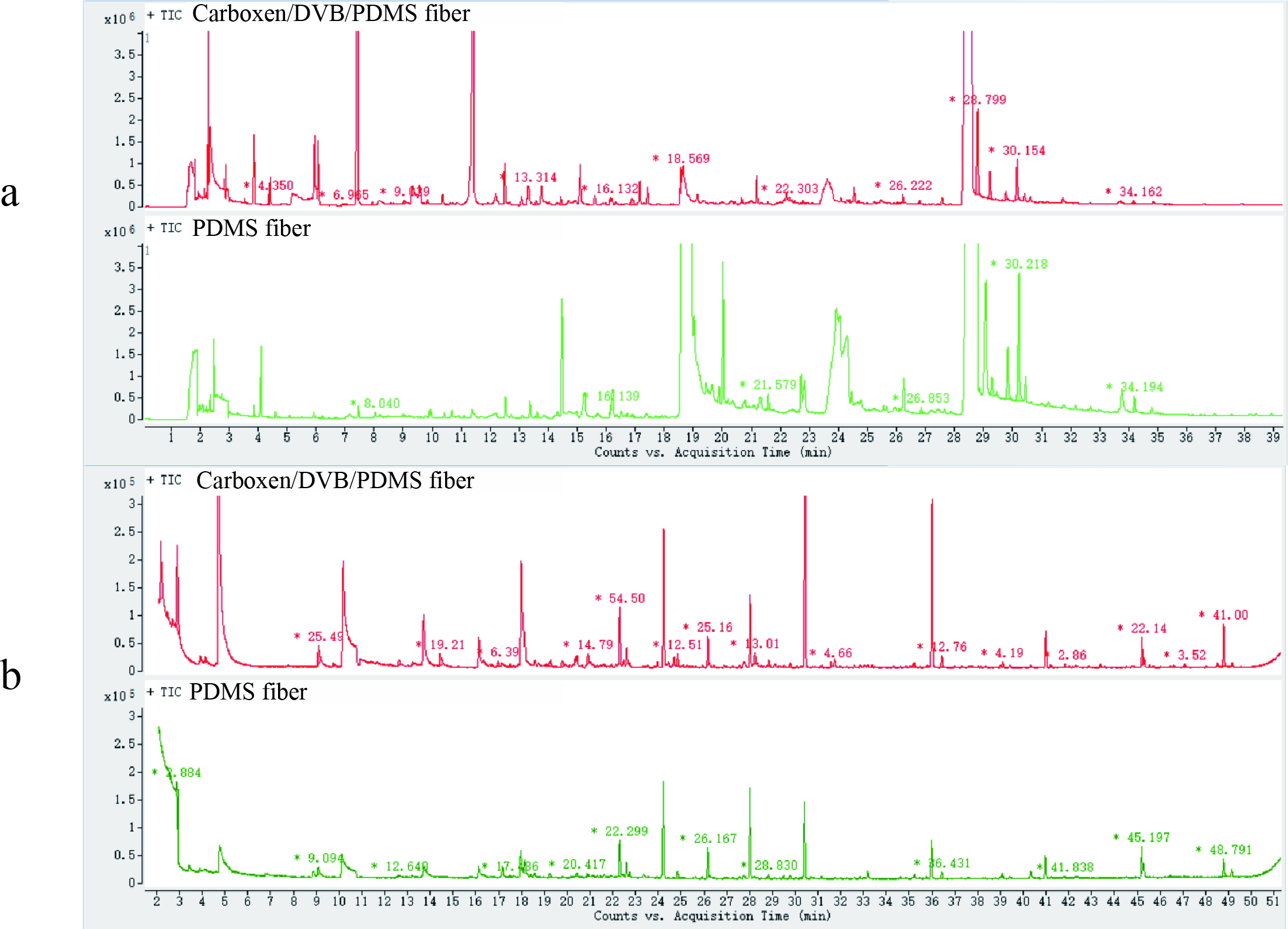

Supplement: Supplementary file 1 [file foods-11-00684-s001.zip › Supplementary documents/Figure S2 the comparation of polar and nonpolar fibers 1.tif]

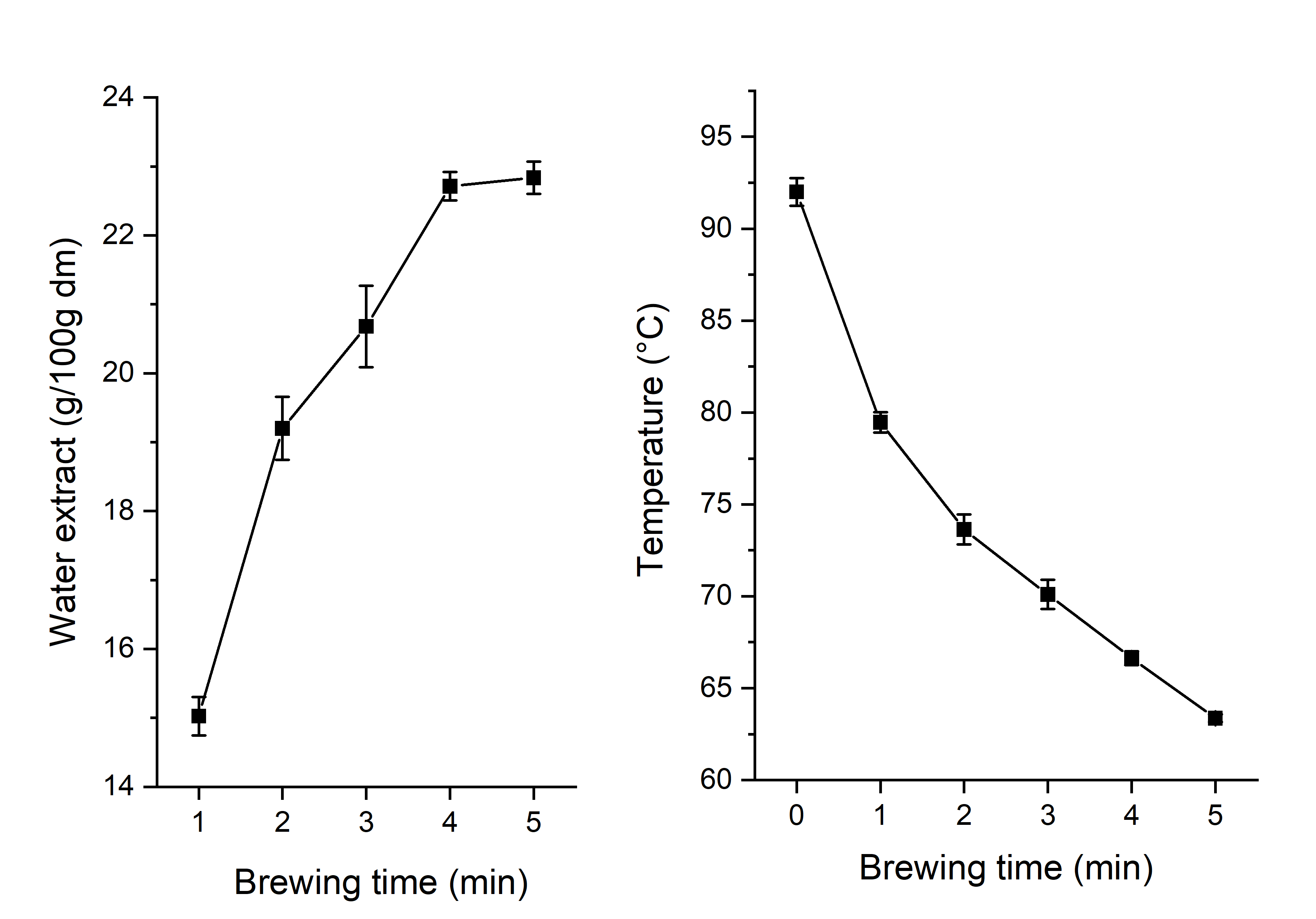

Supplement: Supplementary file 1 [file foods-11-00684-s001.zip › Supplementary documents/Figure S3 The variation of temperature and water during the tea brewing.tif]

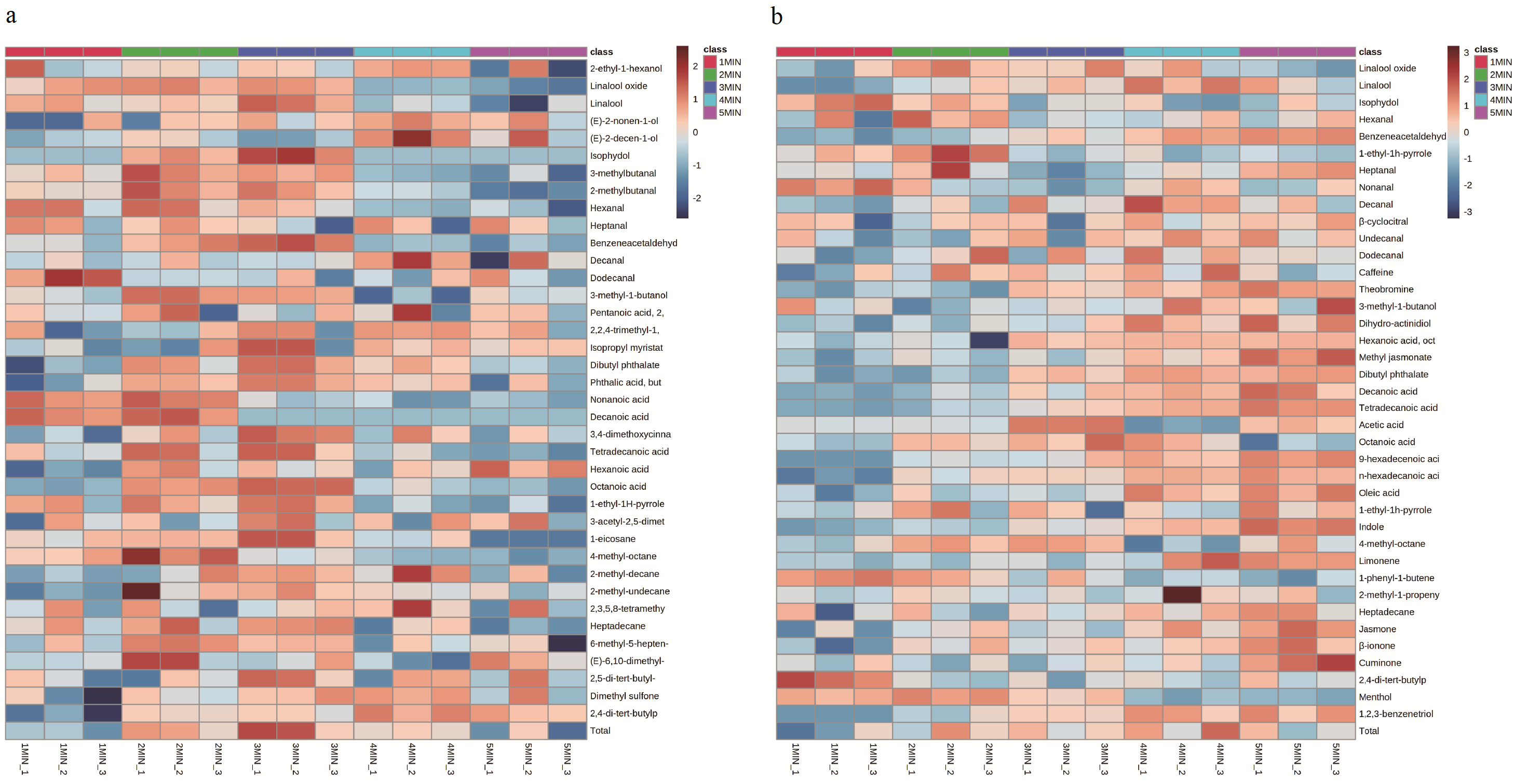

Supplement: Supplementary file 1 [file foods-11-00684-s001.zip › Supplementary documents/Figure S4 The dynamic changes of VOCs during the different brewing time shown by heatmaps.tif]

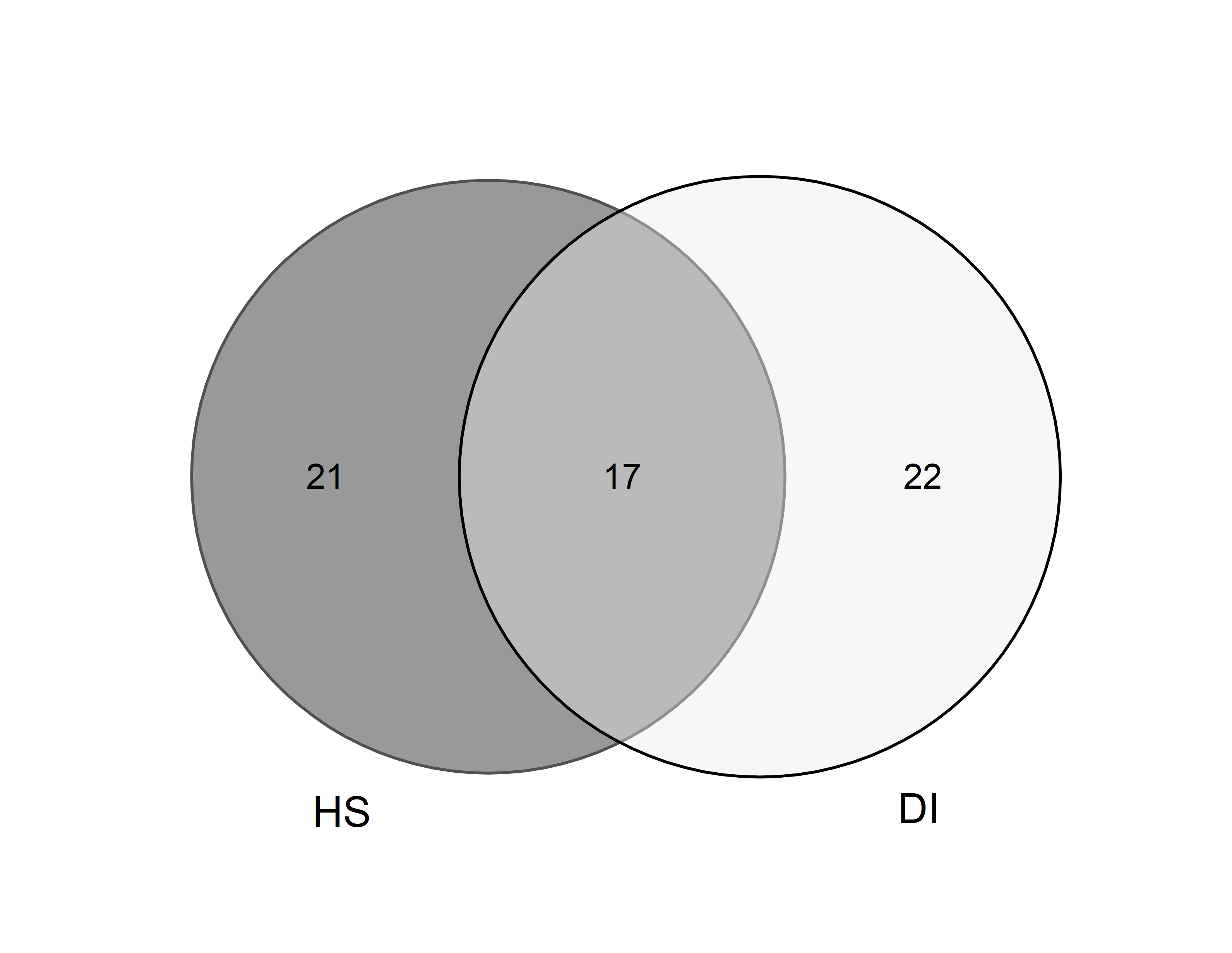

Supplement: Supplementary file 1 [file foods-11-00684-s001.zip › Supplementary documents/Figure S5 Common compounds in HS and DI.tif]
